# Esthetic Reconstruction of Badly Mutilated Endodontically Treated Teeth Using Glass Fiber Reinforced Post: A Case Report

**DOI:** 10.7759/cureus.27662

**Published:** 2022-08-04

**Authors:** Jay Bhopatkar, Anuja Ikhar, Pradnya Nikhade, Manoj Chandak, Anant Heda

**Affiliations:** 1 Department of Conservative Dentistry and Endodontics, Sharad Pawar Dental College and Hospital, Datta Meghe Institute of Medical Sciences, Wardha, IND; 2 Department of Conservative Dentistry and Endodontics, Dr. Rajesh Ramdasji Kambe Dental College and Hospital, Akola, IND

**Keywords:** post and core system, oral rehabilitation, glass fiber post, intracanal post, esthetic restoration

## Abstract

Cast-metal posts have historically been used to repair teeth that have undergone endodontic treatment before receiving aesthetic rehabilitation. Resources have been used to create biocompatible dental posts that can meet functional and cosmetic standards since early posts were difficult to use. These activities led to the development of glass-fiber and carbon-based posts for use in adhesive dental procedures that have translucencies and flexibility close to that of dentin. The use of translucent glass-fiber posts for the repair of pulpless teeth is demonstrated in this case study.

## Introduction

Because it determines the tooth's long-term prognosis, the restoration of the endodontically treated tooth is a crucial consideration during treatment planning [1]. As a result of past restorations, endodontic access preparation, trauma, and dental caries, the pulpless tooth is typically accompanied by a significant loss of coronal and radicular tooth structure [2]. It is universally believed that this loss of hard tissue results in a decreased endodontically treated tooth's ability to transport stresses [3]. Posts are therefore advised for endodontically treated teeth that are very brittle due to poor coronal tooth structure [4-6]. Historically, metal was used to construct prefabricated posts, which can occasionally be seen via the structure of endodontically treated teeth, especially in the anterior area [7]. Loaded with lateral stresses, metal posts appear to rattle at high frequencies due to their high stiffness [8]. These stresses may concentrate in unforeseen "critical areas," which may result in longitudinal root fractures or metal corrosion [9, 10] and ultimately cause tooth loss [11-13]. According to some studies [14, 15], the fact that these metallic materials have substantially greater elasticity moduli than the supporting dentin, this mismatch causes stress to build up in the luting cement, which could cause it to disintegrate. This prompted researchers to look for a plastic-based material with a modulus that is more similar to that of dentin [16].

In 1990, Duret et al. introduced a carbon fiber post as one of the several prefabricated fiber post-and-core systems [17] to lower the post-retained restored tooth failure rate. These relatively recent posts have a special quality known as "anisotropic behavior," in which the substance has distinctive physical properties when loaded in various orientations. They are made of uniformly spaced carbon fibers that are bonded to an epoxy resin matrix. This property may significantly lower the risk of root fracture and decementation, making it relevant to clinical settings [11]. The goal is to develop a "cement-post-core" system that resembles tooth tissues physically and has uniform qualities [18, 19]. Additionally, posts made of quartz and glass fiber that are encased in a resin matrix have been created to meet aesthetic standards [20]. Additionally, restoring endodontically treated teeth with materials that are metal-free, physicochemically homogenous, and have physical qualities comparable to dentin has grown to be a top priority in dentistry [21].

A glass fiber reinforced post's improved light transmission through the root and its surrounding gingival tissues provides an aesthetic benefit. Additionally, fiber-reinforced posts avoid the corrosive reactions problems that prefabricated metal alloy posts could have. The ease of removal of fiber-reinforced posts in the event that endodontic retreatment is necessary is another benefit [21].

This case report outlines a step-by-step procedure for restoring permanent maxillary incisors that have been damaged due to trauma by glass fiber reinforced intracanal post.

## Case presentation

**Figure 1:**
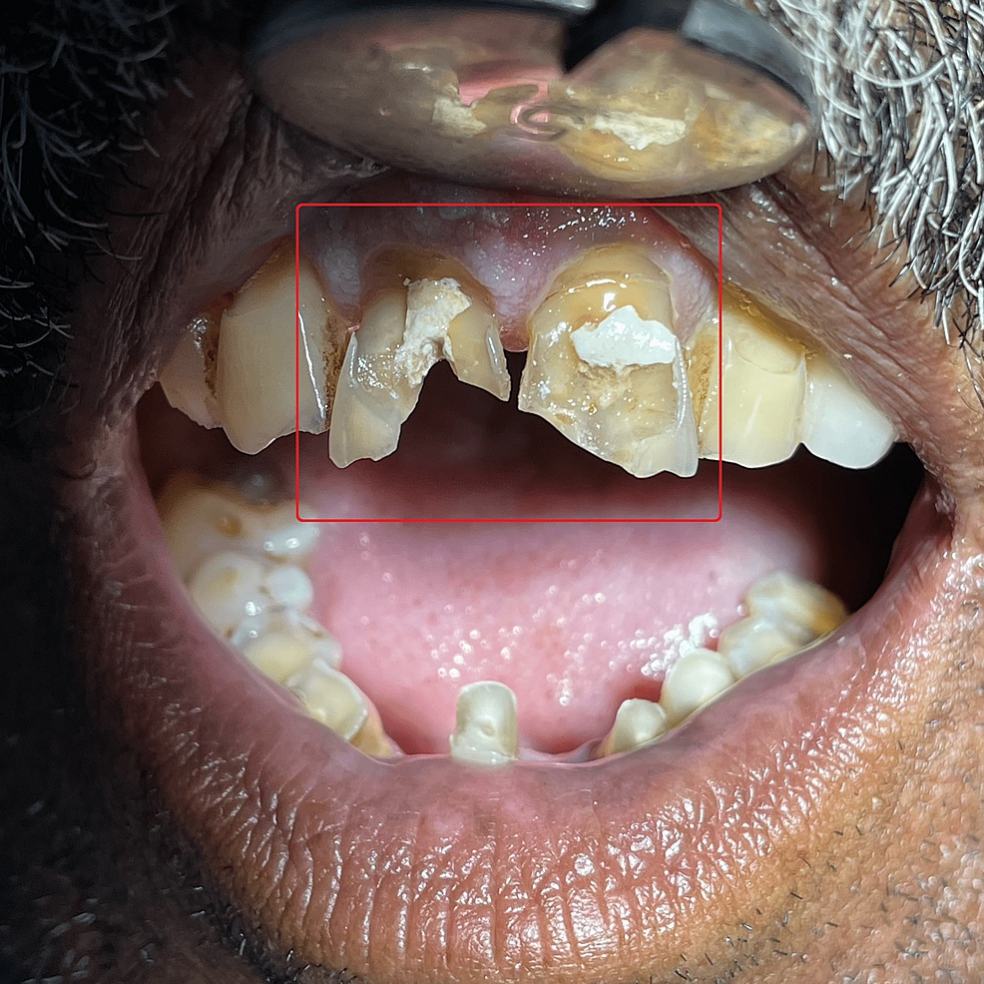
Pre-operative clinical photograph 1

**Figure 2:**
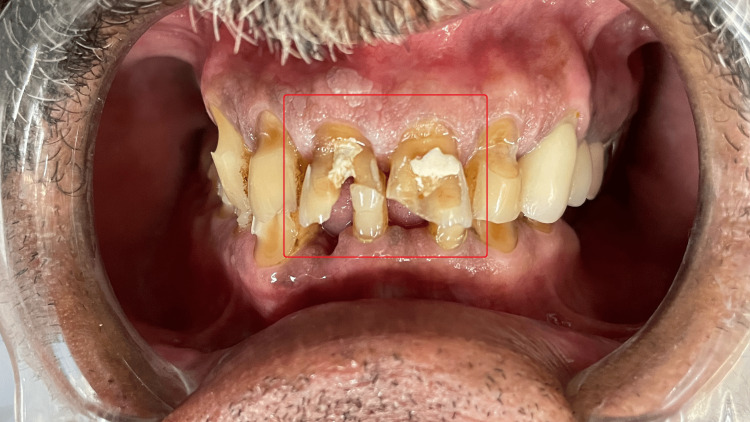
Pre-operative clinical photograph 2

A 57-year-old male patient reported to the Department of Conservative Dentistry and Endodontics of Sharad Pawar Dental College and Hospital, Wardha, Maharashtra, with aesthetic concerns related to dental cracks induced due to a fall (Figure 1 and Figure 2). There was no relevant medical history associated systemically. An intraoral examination revealed unsatisfactory oral hygiene and the absence of caries.

**Figure 3:**
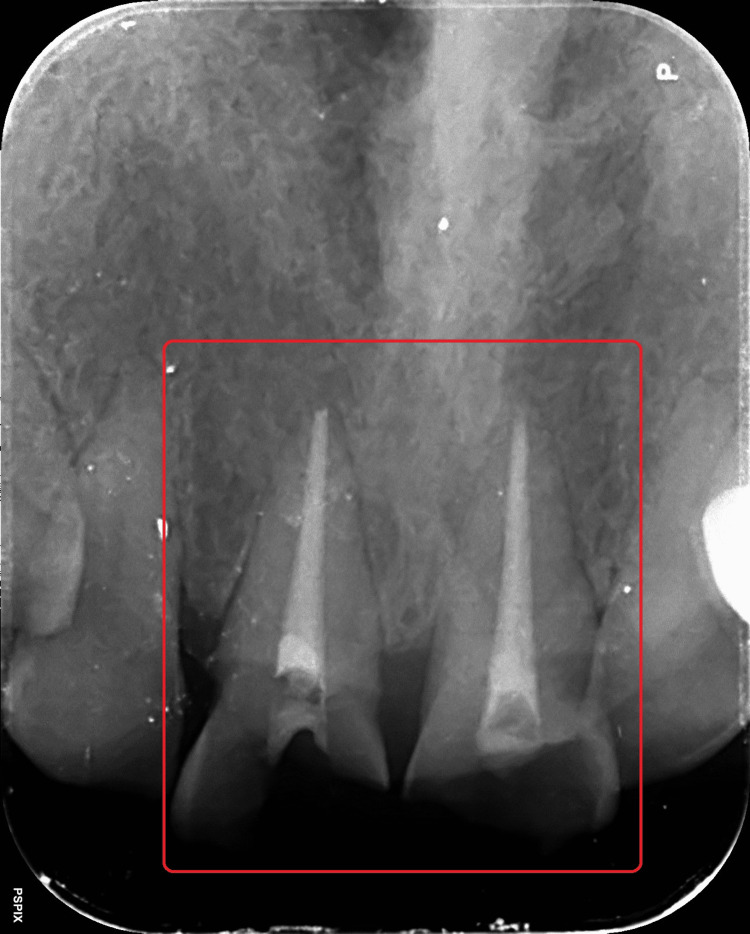
Diagnostic IOPA with respect to teeth 11 and 21 IOPA: Intraoral Periapical Radiograph

The patient had horizontal and oblique crown fractures in teeth 11 and 21, and both were restored with a tooth-colored temporary restorative material. Past dental history revealed that the patient had received conventional root canal therapy for teeth 11 and 21 from a private dental office seven days prior. Radiographic evaluation revealed both teeth 11 and 21 are endodontically treated, with the periradicular tissue remaining healthy and showing no signs of disease or periodontal ligament (PDL) widening (Figure 3). There was no tenderness on percussion with both the teeth. While tooth 11 was severely destructed and required further reinforcing to support an aesthetic crown prosthesis, tooth 21 has enough hard tissue mass to support a crown prosthesis. It was therefore decided to employ a glass fiber reinforced post with composite core to be built onto tooth 11 and a direct composite laminate to be constructed upon tooth 21, followed by the fabrication and cementation of a ceramic crown prosthesis.

Treatment

**Figure 4:**
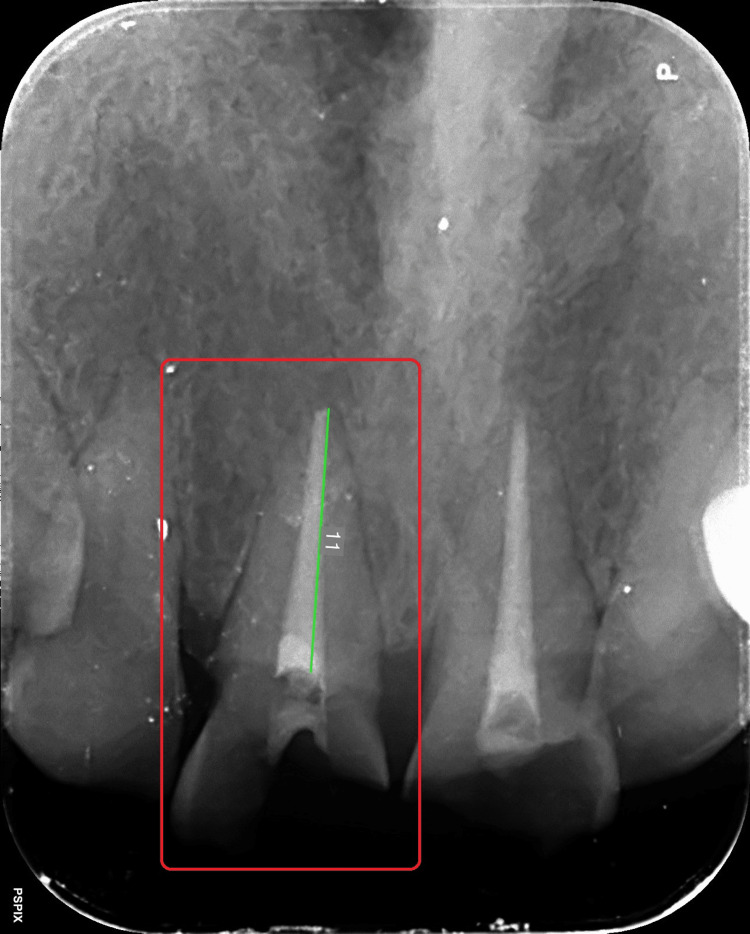
Digitally obtained dimensions of obturation with respect to tooth 11

An informed consent was taken from the patient and the treatment was initiated by removing the tooth-colored temporary restoration from the coronal aspect of both teeth 11 and 21. The dimensions of the obturating material in the canal were then measured in relation to tooth 11 (Figure 4).

**Figure 5:**
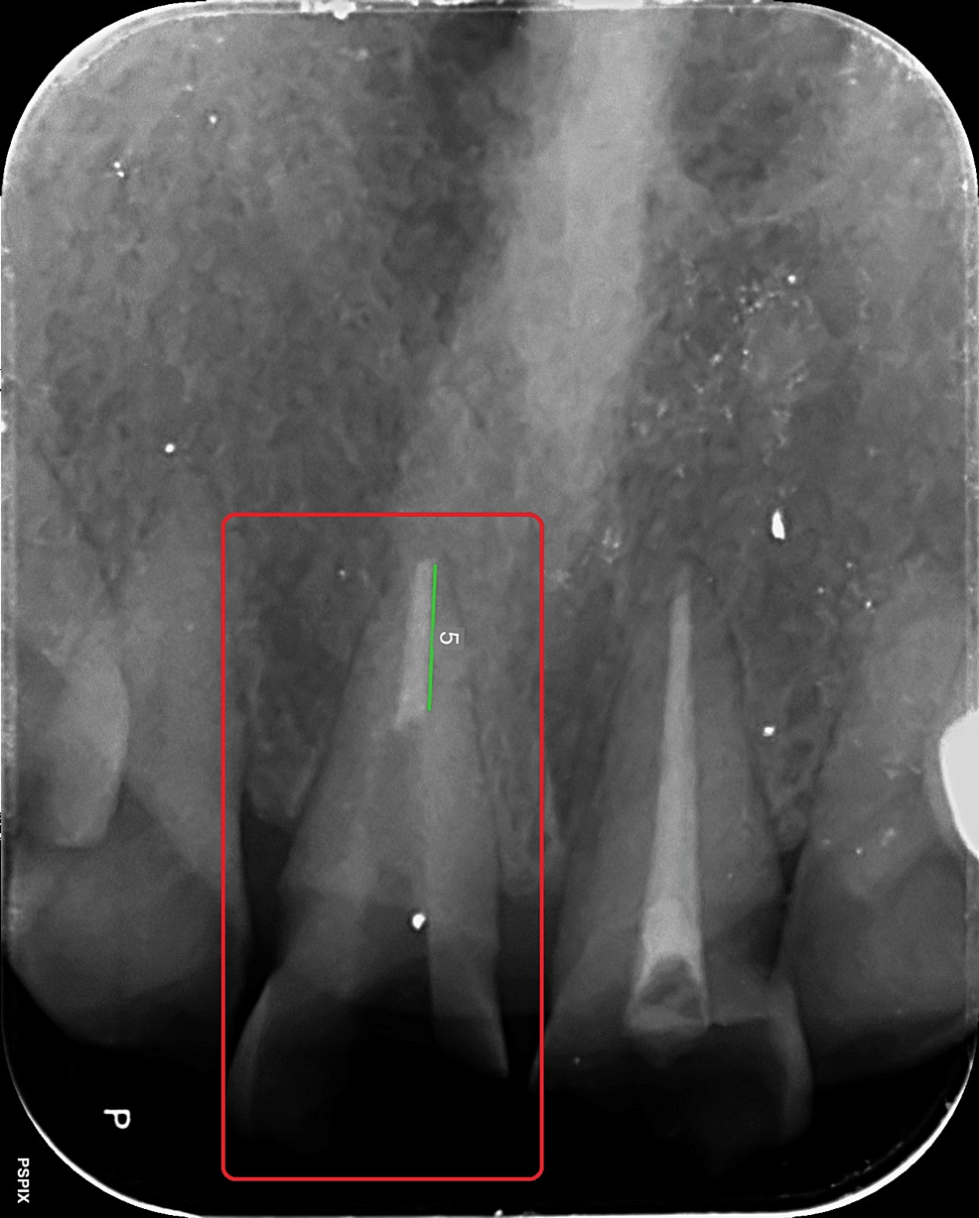
Post space preparation with respect to tooth 11

**Figure 6:**
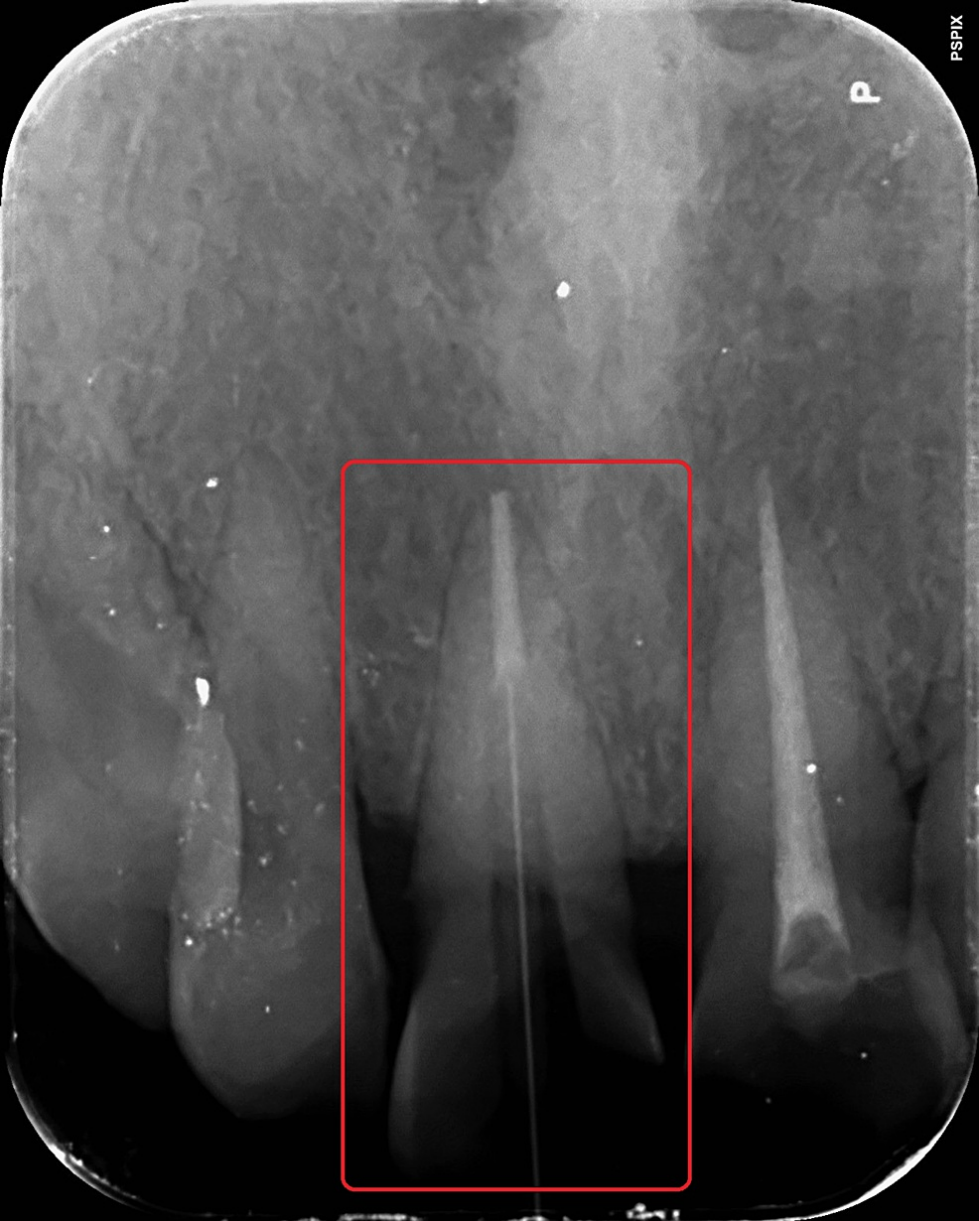
Post fit evaluation with respect to tooth 11

To establish the post space, the canal was consecutively drilled using peso reamers #1 through #4 (MANI Inc., Japan) until only 5 mm of the apical plug remained in the canal's most apical region (Figure 5). Following the establishment of the post space, the post fit was assessed clinically and radiographically (Figure 6). Size 2 posts were chosen (Angelus REFORPOST, Brazil).

**Figure 7:**
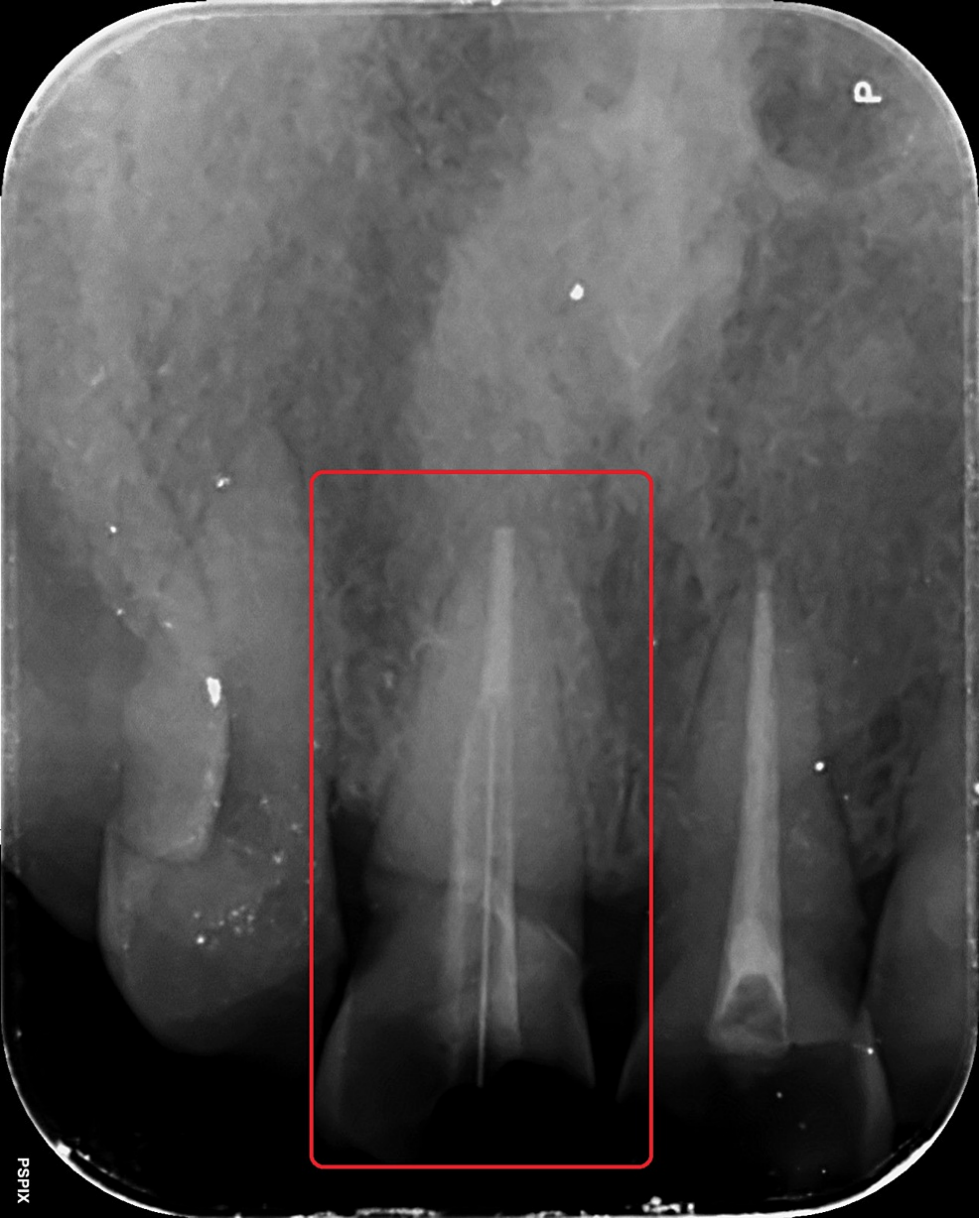
Post cementation with respect to tooth 11

Both the canal of tooth 11 and glass fiber reinforced post were etched (37% Phosphoric acid, Prime Dental, India) respectively for 30 seconds. The canal and post were then cleaned and allowed to air dry. Following the manufacturer's recommendations, bonding agent (3M ESPE Adper Single Bond 2; 3M, Saint Paul, MN, USA) application and ultraviolet light curing was performed. After which, the placement of flowable composites (Calibra Universal self-adhesive resin cement, Dentsply Sirona, Charlotte, NC, USA) in the post space is done, and then the post was introduced with composite inside. Both were then light-cured for 60 seconds (Figure 7).

Using a round bur (BR-45, MANI Inc., Japan), excess post length was sheared off. The remaining coronal crown portions of 11 and 12 were then acid-etched, cleaned, and UV-bonded. In the last stage of the treatment, direct composite laminate (Spectrum Microhybrid Composite resin, Dentsply Sirona, Charlotte, NC, USA) was constructed over 12, while a micro-hybrid composite core (Spectrum Microhybrid Composite resin, Dentsply Sirona, Charlotte, NC, USA) was built over 11.

**Figure 8:**
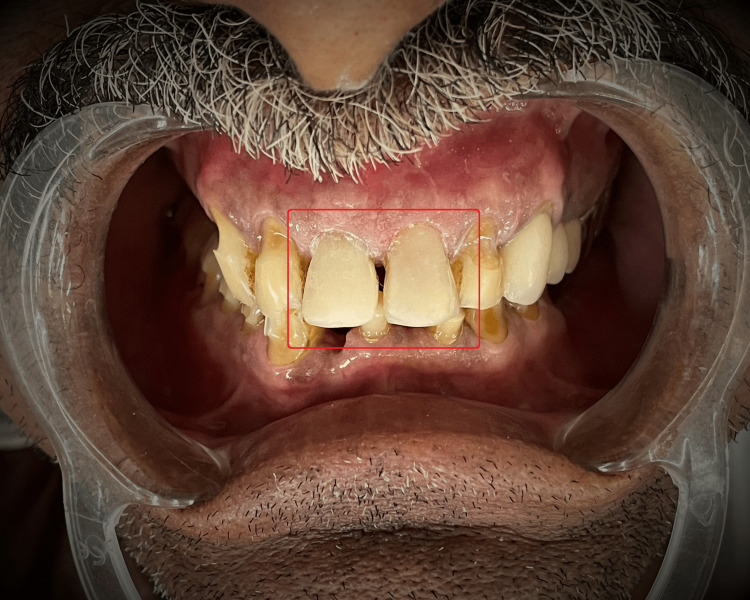
Post-operative clinical photograph 1

**Figure 9:**
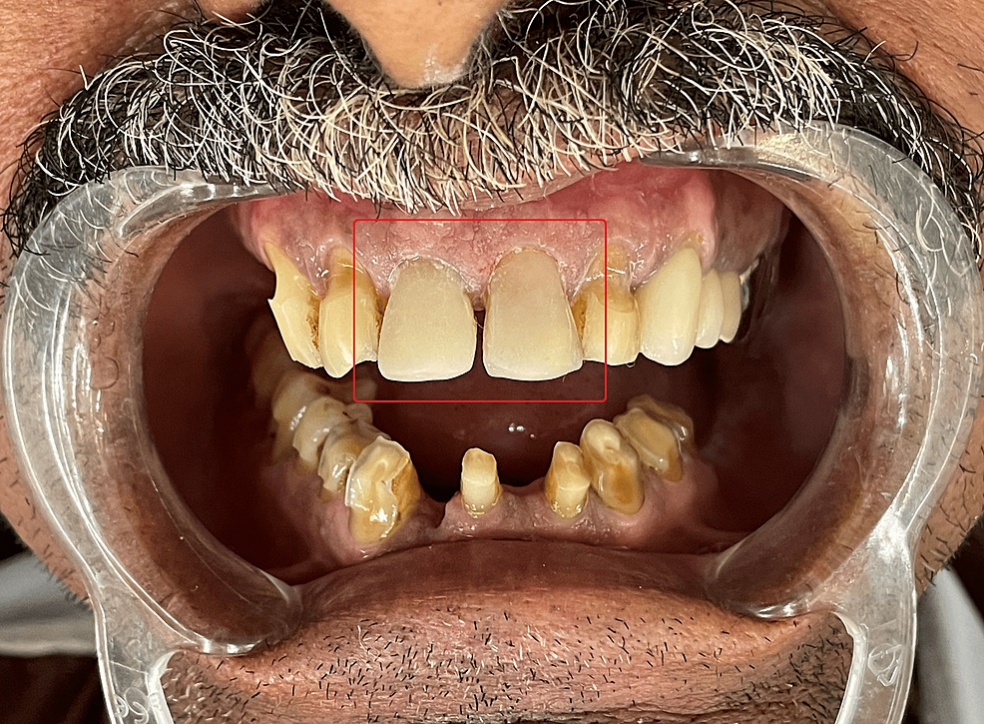
Post-operative clinical photograph 2

Abrasive polishing paper discs (Shofu Super Snap Mini kit, Japan) were used for finishing, polishing, and final adjustments (Figure 8 and Figure 9).

## Discussion

Adults may bite with a maximum force of 90 KGF and chew with a force ranging from 7 to 15 KGF [22]. For the restored tooth to be clinically effective, it must be resilient to these stresses over time. As a result, the post-core system must resemble dentin and have a proper stress distribution along the root. Less stress is transferred from the post to the dentin when a fiber post used for restoration has an elastic modulus similar to intraradicular dentin [23].

According to reports, the elasticity of fiber-reinforced composite posts (FRCP) is comparable to that of dentin [21, 24]. Dentinal bonding is thought to create a mono-block dentin-post-core system, which would improve the force distribution along the root under stress [25].

In this case report, the glass fibers were used to modify the post system, which was developed initially for tooth-colored fillings and fixed partial dentures (FPDs). Unidirectional R-glass (65% weight), Bisphenol A-glycidyl methacrylate/Dodecane dimethacrylate/Urethane dimethacrylate/Triethylene glycol dimethacrylate matrix and Silicon dioxide filler particles (3.5% weight) make up this substance. The placement and orientation of various types of fibers can affect the fracture stress of FRCP, according to Dyer et al. [26]. They showed how the test specimen seems to have unidirectional glass fiber reinforcement, which increases the flexural rigidity and, thereby, the load of breakdown of the post.

Greater flexural strength, ease of handling, applications in high stress-bearing regions, aesthetically acceptable, and the capacity to attach to any composite are just a few of the benefits that glass fiber posts have over other posts [27]. The current study demonstrates excellent retention within the canal for a considerable amount of time. For badly mutilated anterior teeth, these reasons make this technique preferred over other types of posts.

There is better stress distribution between the post and dentin because the glass fiber post's modulus of elasticity (13-40 GPa) is equivalent to dentin's (15-25 GPa), which enhances the pliability of teeth while under stress. Fiber posts help to reduce the likelihood of irreparable root fractures [28, 29].

Resin adhesive cement is the cement utilized for the cementation of glass fiber posts. Employing an adhesive bonding strategy, this cement forms a firm fusion between the post and the core as well as to the remaining tooth structure. As a result, there is a more effective transfer and dispersion of the functional stresses from the bonding surface to the tooth [30]. Adhesive technique integration produced a "monobloc" type of repair by combining post and core processes [19]. Using a resin adhesive cement system with a glass fiber post and core, a "monobloc" kind of repair is made in order to more efficiently transfer and distribute functional stresses throughout the tooth.

## Conclusions

The adoption of specialized unidirectional glass fiber posts, with the modelled internal architecture of the root canal of this case, demonstrated satisfactory clinical and radiographic characteristics. Great light conductivity, biocompatibility, ease of handling, equivalent elasticity modulus to that of dentin, flexural and fatigue strength, and excellent cosmetic qualities are all inherent in these posts. This procedure is suited for repairing teeth that have had endodontic treatment and is efficient, minimally invasive, and appropriate.

## References

[1] (2009). Evidence-based review of clinical studies on restorative dentistry. J Endod.

[2] Mannocci F, Bertelli E, Sherriff M, Watson TF, Ford TR (2002). Three-year clinical comparison of survival of endodontically treated teeth restored with either full cast coverage or with direct composite restoration. J Prosthet Dent.

[3] Naumann M, Blankenstein F, Dietrich T (2005). Survival of glass fibre reinforced composite post restorations after 2 years-an observational clinical study. J Dent.

[4] Sorensen JA, Martinoff JT (1984). Intracoronal reinforcement and coronal coverage: a study of endodontically treated teeth. J Prosthet Dent.

[5] Assif D, Gorfil C (1994). Biomechanical considerations in restoring endodontically treated teeth. J Prosthet Dent.

[6] Morgano SM (1996). Restoration of pulpless teeth: application of traditional principles in present and future contexts. J Prosthet Dent.

[7] Abo El-Ela OA, Atta OA, El-Mowafy O (2008). Fracture resistance of anterior teeth restored with a novel nonmetallic post. J Can Dent Assoc.

[8] Malferrari S, Monaco C, Scotti R (2003). Clinical evaluation of teeth restored with quartz fiber-reinforced epoxy resin posts. Int J Prosthodont.

[9] Goodacre CJ, Spolnik KJ (1994). The prosthodontic management of endodontically treated teeth: a literature review. Part I. Success and failure data, treatment concepts. J Prosthodont.

[10] Ozkurt Z, Işeri U, Kazazoğlu E (2010). Zirconia ceramic post systems: a literature review and a case report. Dent Mater J.

[11] Trabert KC, Cooney JP (1984). The endodontically treated tooth. Restorative concepts and techniques. Dent Clin North Am.

[12] Morgano SM, Milot P (1993). Clinical success of cast metal posts and cores. J Prosthet Dent.

[13] Duret B, Reynaud M, Duret F (1990). New concept of coronoradicular reconstruction: the Composipost (1) [Article in French]. Chir Dent Fr.

[14] King PA, Setchell DJ, Rees JS (2003). Clinical evaluation of a carbon fibre reinforced carbon endodontic post. J Oral Rehabil.

[15] Brown D (2000). Fibre-reinforced materials. Dent Update.

[16] Preethi G, Kala M (2008). Clinical evaluation of carbon fiber reinforced carbon endodontic post, glass fiber reinforced post with cast post and core: a one year comparative clinical study. J Conserv Dent.

[17] Duret B, Reynaud M, Duret F (1990). A new concept of corono-radicular reconstruction, the Composipost (2) [Article in French]. Chir Dent Fr.

[18] Wagnild GW, Mueller KI (2006). The restoration of endodontically treated tooth. In: Pathways of the Pulp.

[19] Cormier CJ, Burns DR, Moon P (2001). In vitro comparison of the fracture resistance and failure mode of fiber, ceramic, and conventional post systems at various stages of restoration. J Prosthodont.

[20] Gutmann JL (1992). The dentin-root complex: anatomic and biologic considerations in restoring endodontically treated teeth. J Prosthet Dent.

[21] Akkayan B, Gülmez T (2002). Resistance to fracture of endodontically treated teeth restored with different post systems. J Prosthet Dent.

[22] Salameh Z, Sorrentino R, Ounsi HF, Goracci C, Tashkandi E, Tay FR, Ferrari M (2007). Effect of different all-ceramic crown system on fracture resistance and failure pattern of endodontically treated maxillary premolars restored with and without glass fiber posts. J Endod.

[23] Pierrisnard L, Bohin F, Renault P, Barquins M (2002). Corono-radicular reconstruction of pulpless teeth: a mechanical study using finite element analysis. J Prosthet Dent.

[24] Qualtrough AJ, Mannocci F (2003). Tooth-colored post systems: a review. Oper Dent.

[25] Sirimai S, Riis DN, Morgano SM (1999). An in vitro study of the fracture resistance and the incidence of vertical root fracture of pulpless teeth restored with six post-and-core systems. J Prosth Dent.

[26] Dyer SR, Lassila LV, Jokinen M, Vallittu PK (2004). Effect of fiber position and orientation on fracture load of fiber-reinforced composite. Dent Mater.

[27] Babaji P (2015). Crowns in Pediatric Dentistry. New Delhi: Jaypee Brothers Medical Publishers.

[28] Newman MP, Yaman P, Dennison J, Rafter M, Billy E (2003). Fracture resistance of endodontically treated teeth restored with composite posts. J Prosthet Dent.

[29] Bitter K, Kielbassa AM (2007). Post-endodontic restorations with adhesively luted fiber-reinforced composite post systems: a review. Am J Dent.

[30] Mohammadi N, Kahnamoii MA, Yeganeh PK, Navimipour EJ (2009). Effect of fiber post and cusp coverage on fracture resistance of endodontically treated maxillary premolars directly restored with composite resin. J Endod.

